# Influence of circulatory shock at hospital admission on outcome after out-of-hospital cardiac arrest

**DOI:** 10.1038/s41598-022-12310-5

**Published:** 2022-05-18

**Authors:** Joachim Düring, Martin Annborn, Josef Dankiewicz, Allison Dupont, Sune Forsberg, Hans Friberg, Karl B. Kern, Teresa L. May, John McPherson, Nainesh Patel, David B. Seder, Pascal Stammet, Kjetil Sunde, Eldar Søreide, Susann Ullén, Niklas Nielsen

**Affiliations:** 1 Department of Clinical Sciences, Anesthesia & Intensive Care, Lund University, Skåne University Hospital, Malmö, Sweden; 2 Department of Clinical Sciences Lund, Anesthesia & Intensive Care, Lund University, Helsingborg Hospital, Helsingborg, Sweden; 3 Department of Clinical Sciences, Cardiology, Lund University, Skåne University Hospital, Lund, Sweden; 4 Department of Cardiology, Northside Cardiovascular Institute, Atlanta, GA USA; 5 Department of Intensive Care, Norrtälje Hospital, Karolinska Institute, Norrtälje, Sweden; 6 Center for Resuscitation Science, Karolinska Institute, Stockholm, Sweden; 7 Division of Cardiology Department of Medicine, University of Arizona, Tucson, AZ USA; 8 Department of Critical Care Services, Maine Medical Center, Portland, ME USA; 9 Vanderbilt University Medical Center, Nashville, USA; 10 Department of Cardiology, Lehigh Valley Health Network, Allentown, PA USA; 11 Department of Intensive Care Medicine, Centre Hospitalier de Luxembourg, Luxembourg City, Luxembourg; 12 Faculty of Science, Technology and Medicine, University of Luxembourg, Esch-sur-Alzette, Luxembourg; 13 Department of Anesthesiology, Division of Emergencies and Critical Care, Oslo University Hospital, Oslo, Norway; 14 Institute of Clinical Medicine, University of Oslo, Oslo, Norway; 15 Critical Care and Anesthesiology Research Group, Stavanger University Hospital, Stavanger, Norway; 16 Department of Clinical Medicine, University of Bergen, Bergen, Norway; 17 Clinical Studies Sweden- Forum South, Skåne University Hospital, Lund, Sweden

**Keywords:** Ventricular fibrillation, Ventricular tachycardia, Heart failure, Hypoxic-ischaemic encephalopathy, Disorders of consciousness

## Abstract

Hypotension after cardiac arrest could aggravate prolonged hypoxic ischemic encephalopathy. The association of circulatory shock at hospital admission with outcome after cardiac arrest has not been well studied. The objective of this study was to investigate the independent association of circulatory shock at hospital admission with neurologic outcome, and to evaluate whether cardiovascular comorbidities interact with circulatory shock. 4004 adult patients with out-of-hospital cardiac arrest enrolled in the International Cardiac Arrest Registry 2006–2017 were included in analysis. Circulatory shock was defined as a systolic blood pressure below 90 mmHg and/or medical or mechanical supportive measures to maintain adequate perfusion during hospital admission. Primary outcome was cerebral performance category (CPC) dichotomized as good, (CPC 1–2) versus poor (CPC 3–5) outcome at hospital discharge. 38% of included patients were in circulatory shock at hospital admission, 32% had good neurologic outcome at hospital discharge. The adjusted odds ratio for good neurologic outcome in patients without preexisting cardiovascular disease with circulatory shock at hospital admission was 0.60 [0.46–0.79]. No significant interaction was detected with preexisting comorbidities in the main analysis. We conclude that circulatory shock at hospital admission after out-of-hospital cardiac arrest is independently associated with poor neurologic outcome.

## Introduction

The main driver of poor long-term outcome after out-of-hospital cardiac arrest (OHCA) is hypoxic ischemic encephalopathy (HIE)^1^. The early phase after cardiac arrest can be complicated by circulatory shock, due to low systemic vascular resistance and/or myocardial dysfunction^2^, potentially aggravating HIE through prolonged cerebral hypoperfusion. The reported incidence of circulatory shock at presentation after OHCA is 15–68%^3–6^. In studies circulatory shock is defined as hypotension, clinical signs of hypoperfusion, or the need for supportive measures to maintain an acceptable perfusion pressure. The lack of a universally accepted definition of circulatory shock and differences in patient selection could explain the large variation in the incidence reported. Outcomes after cardiac arrest include survival and/or neurologic recovery, dichotomized as favorable or poor, according to the International Liaison Committee of Resucitation (ILCOR)^7^. Observational studies have failed to prove an association between increased cardiac output post-cardiac arrest and improved long-term outcomes^8–11^. Current guidelines based on observational studies recommend a mean arterial pressure (MAP) above 65 mmHg during the initial phase of critical care^12,13^. Impaired autoregulation of cerebral perfusion has been reported in 35% of post resuscitation patients and is overrepresented in patients with preexisting arterial hypertension^14^. This may explain lack of beneficial effects in interventional trials targeting increased blood pressure for patients with hypotension post OHCA^15,16^. Despite the many studies on the association between circulatory shock on admission and outcome after OHCA the data are conflicting^17–19^. In this observational study of a large international cardiac arrest database, our hypothesis was that circulatory shock at hospital admission after OHCA is independently associated with neurologic outcome. As a secondary analysis, we explored the relative contribution of circulatory shock in patients with cardiovascular comorbidities, on neurologic outcome.

## Methods

### Study design and setting

This is a retrospective registry study of patients enrolled from 2006 to 2017 in The International Cardiac Arrest Registry (INTCAR), a large North American/European post cardiac arrest registry containing a defined core dataset. Ethical Review Boards (ERB) in each country approved data collection and participation. Informed consent was waived from all participants in line with the Helsinki declaration, by approval of the Ethical Review Board in Lund, reference number: REPN Lund Dnr 2007/272.

### Study population

All patients in the INTCAR registry were screened for eligibility. Exclusion criteria were the following: in-hospital cardiac arrest (IHCA); Patients awake on admission defined as Glasgow Coma Score motor component equal to 6; Age below 18 years; Missing records on the presence or absence of circulatory shock on admission or Cerebral Performance Category (CPC) at hospital discharge. Subgroup analysis was performed in patients with continuous explanatory variables linearly associated with outcome.

### Outcomes and definitions

According to the International Liaison Committee of Resucitation, core outcome recommendations^20^, the primary outcome was dichotomized as good (CPC 1–2) or poor (CPC 3–5) at hospital discharge. The CPC scale ranges from 1 to 5, with 1 representing good cerebral performance or minor disability, 2 moderate disability, 3 severe disability, 4 coma or vegetative state, and 5 brain death^21^. By default, poor neurologic recovery according to this definition, is correlated with mortality. Circulatory shock was defined as systolic blood pressure < 90 mm Hg and/or the need for supportive measures, such as inotropes, vasoactive drugs, mechanical circulatory support devices to maintain a systolic blood pressure ≥ 90 mmHg or end-organ hypoperfusion. Hospital admission was defined as the first unit the patient presented at after the OHCA, emergency department (ED), the intensive Care Unit (ICU) or coronary angiography lab, depending on the routines of the including hospital. Time to start of advanced life support (ALS) was defined as time from witnessed cardiac arrest or, in the event of unwitnessed arrest, time from emergency call to start of advanced life support by medical personnel. Return of spontaneous circulation (ROSC) was defined to have occurred when chest compressions were not required for 20 consecutive minutes and signs of circulation persisted. Predefined co-morbidities were registered if they involved current pharmacological or prior surgical treatment or were under active medical supervision at the time of arrest. Obesity was defined as body mass index > 35.

### Statistical analysis

Continuous descriptive data are presented as medians with interquartile (IQR) range. Differences in baseline variables were tested using Chi-square test for categorical data, while continuous data were tested using Student’s t-Test or Mann–Whitney test as appropriate. Variables with less than 20% missing were considered as candidates for explanatory variables in analyses. Pairwise correlation was estimated for all combinations of variables. The least relevant variable, from the perspective of our main hypothesis, was dropped from analysis if Pearson correlation coefficient was over 0.75 or less than -0.75. Skewed continuous explanatory variables were transformed to normal distribution, choosing the method yielding the lowest Pearson P statistic/degrees of freedom^22^. All continuous variables were scaled to standard deviations (SD) and centered^22^. Analysis of variables with more than 5% missing did not reveal any evidence for systemic mechanism of missingness. Final analyses were based on the pooled estimates, using Rubin’s rules, from 24 datasets imputed by chained equations with predictive mean matching for continuous variables and logistic regression for categorical data, using all modelled variables and outcomes. Imputation was done under the assumption of missing at random. The association between circulatory shock and outcome was estimated using generalized additive methods (GAM) with smooth functions fitting continuous data to cubic restricted splines using 5 knots. Fit of the smooth functions for continuous data were visually checked by plotting residuals for the explanatory variables versus the log odds of the dependent variable, and by estimating the k-index. Models were adjusted for: first monitored rhythm: Pulseless Electrical Activity (PEA), asystole or either pulseless ventricular tachycardia or ventricular fibrillation (VT/VF); Presence of ST-Elevation Myocardial Infarction (yes/no); witnessed arrest (yes/no); bystander cardiopulmonary resuscitation (yes/no); time to start of ALS (square root transformed minutes); sex (male/female); time to ROSC (ordered quantiles transformed minutes); age (ordered quantiles transformed years); circulatory shock on presentation at hospital (yes/no), presence of the following comorbidities(present/not present): Neuro-vascular disease, Chronic obstructive pulmonary disease, obesity, diabetes, chronic kidney disease, hypertension, coronary artery disease, congestive heart failure, arrhythmia; interaction between comorbidities and circulatory shock on presentation. Results from GAM models are presented as odds ratios (OR) with 95% confidence interval (CI). To estimate the association with outcome for the continuous variables in our model, subgroup analysis was performed on a patient group defined by continuous variables linearly associated with neurologic outcome at hospital discharge. Cutoffs for the variables in subgroup analysis was empirically tested and confirmed linear. Complete cases models confirmed concurvity for continuous data < 0.01, and variance inflation index (VIF) for categorical data < 3, indicating no significant concurvity or multicollinearity. Goodness of fit was confirmed with Hosmer–Lemeshow test. Sensitivity analysis was performed comparing our results with that of data imputed with a span of 75–125% of originally imputed using a calibrated-∂ adjustment^23^ data for variables with missingness more than 5%. Because of the exploratory nature of this analysis, no correction was done for multitesting.

## Results

**Figure 1:**
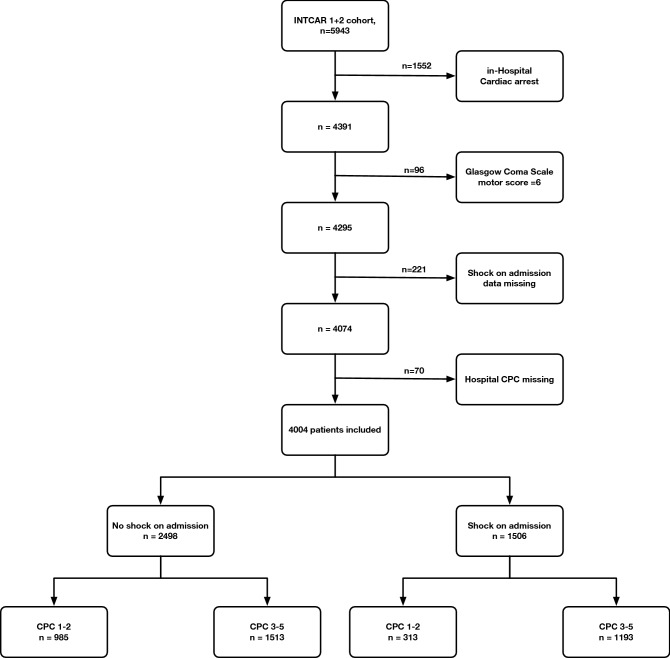
CONSORT flowchart. 4004 patients were included in the final analysis. *CPC* Cerebral Performance Category.

Out of 5943 enrolled patients in the INTCAR registry 4004 were eligible for analysis (Fig. 1). Of the included patients in final analysis, 1506 (38%) patients were in circulatory shock at hospital admission and 1298 (32%) patients had good neurologic outcome (CPC 1–2) at hospital discharge. Circulatory shock was more frequent in women, 42.0 versus 35.6% in men. Patients with circulatory shock at hospital admission were older 64 [54–73] versus 62 [51–72] years, had less witnessed cardiac arrest 75.8 versus 79.4%, higher prevalence of non-shockable rhythm (asystole or PEA) 52.3 versus 47.7%, longer median time to ROSC 29 [18–42] versus 20 [13–30] minutes, higher burden of comorbidities, and presented with worse neurologic clinical status. The use of a mechanical chest compression device was more common in the circulatory shock population, 22.9 versus 19.5% (Table 1).

**Table 1:** Baseline characteristics stratified according to circulatory shock at hospital admission. Patients included in main analysis, n = 4004.

	Circulatory shock	No circulatory shock	% Missing
n	1506	2498	
Male sex (%)	978 (65.1)	1768 (70.9)	0.1
Age, years (median [IQR])	64 [54–73]	62 [51–72]	0.0
**Preexisting comorbidities**			
Coronary artery disease (%)	376 (25.0)	610 (24.4)	0.0
Congestive heart failure (%)	291 (19.3)	397 (15.9)	0.0
Arrhythmia (%)	223 (14.8)	309 (12.4)	0.0
COPD (%)	234 (15.5)	357 (14.3)	0.0
Hypertension (%)	744 (49.4)	1095 (43.8)	0.0
Chronic kidney disease (%)	179 (11.9)	198 (7.9)	0.0
Neurovascular disease (%)	181 (12.0)	304 (12.2)	0.0
Body mass index > 35 (%)	165 (11.0)	232 (9.3)	0.0
Any type of diabetes (%)	371 (24.6)	507 (20.3)	0.0
Previously healthy (%)	262 (17.4)	505 (20.2)	0.0
**First documented rhythm**			
Asystole (%)	406 (28.4)	526 (22.1)	4.9
Pulseless electrical activity (%)	342 (23.9)	437 (18.4)	4.9
Shockable rhythm (%)	681 (47.7)	1415 (59.5)	4.9
**Intra-arrest characteristics**			
Witnessed cardiac arrest (%)	1134 (75.8)	1968 (79.4)	0.7
Bystander CPR (%)	942 (62.8)	1632 (65.7)	0.5
Mechanical chest compressions (%)	340 (22.9)	472 (19.5)	2.5
Time to ALS, minutes (median [IQR])	8 [5–12]	7 [5–11]	10.8
Time to ROSC, minutes (median [IQR])	29 [18–42]	20 [13–30]	5.6
STEMI on ECG (%)	365 (26.1)	554 (23.8)	6.9
GCSm 3–5 at hospital admission (%)	307 (21.0)	652 (26.8)	2.7

Categorical data is presented as absolute counts and frequencies, continuous data as medians with interquartile range. *IQR* interquartile range; *COPD* Chronic obstructive pulmonary disease; *CPR* Cardiopulmonary resuscitation; *ALS* Advanced life support; *ROSC* Return of spontaneous circulation; *STEMI* ST-Elevation myocardial infarction; *ECG* Electrocardiogram; *GCSm* Glasgow coma score motor component.

**Figure 2:**
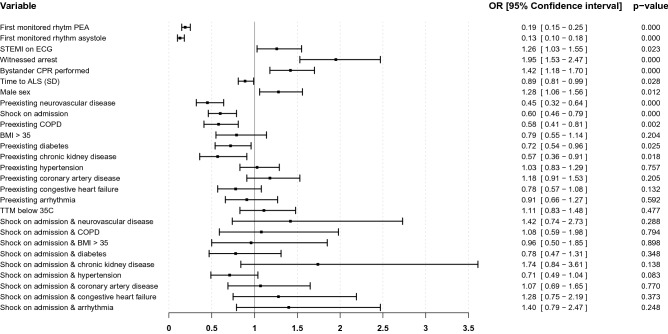
Association with good neurological outcome at hospital discharge. Forest plot illustrating the odds ratios for Cerebral Performance Category 1–2, in a multivariate generalized additive methods model. Analysis was performed in the full cohort, n = 4004. The reference category for first monitored rhythm is shockable rhythm (Ventricular fibrillation or ventricular tachycardia). Time to ALS time to ALS has been square root transformed scaled to standard deviations and centered. Interaction between circulatory shock and preexisting comorbidities are included in model. Point estimates are presented as odds ratios (OR) with 95% confidence intervals. Time to return of spontaneous circulation and age are included in model but not presented due to nonlinearity. *PEA* Pulseless electrical activity; *STEMI* ST-Elevation myocardial infarction; *ECG* Electrocardiogram; *CPR* Cardiopulmonary resuscitation; *SD* Standard deviation; *COPD* Chronic pulmonary obstructive disease; *BMI* Body mass index; *TTM* Targeted temperature management.

The explanatory model revealed a nonlinear association between age, ROSC and outcome. The association with time to ALS was linear (Supplementary Fig. S1). For the full population, the adjusted OR for circulatory shock at hospital admission as an explanatory factor for good neurologic outcome, in the setting of no preexisting comorbidity, was 0.60 [0.46–0.79]. No significant interaction with preexisting comorbidities was detected (Fig. 2). The explained variance of the model was 32.6%.

**Figure 3:**
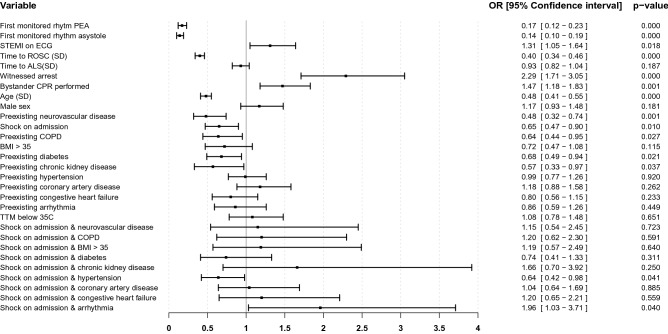
Association with good neurological outcome at hospital discharge in subgroup analysis. Forest plot illustrating the odds ratios for Cerebral Performance Category 1–2, in a multivariate generalized additive methods model. Analysis was performed in a subgroup of patients with linear continuous explanatory variables, aged 42–92 years, with time to ROSC 9–87 min. The reference category for first monitored rhythm is shockable rhythm (Ventricular fibrillation or ventricular tachycardia). Age (years) and time to ROSC (minutes) have been transformed to normality by ordered quantiles, time to ALS has been square root transformed. After transformation, the variables have been scaled to standard deviations and centered. Interaction between circulatory shock and preexisting comorbidities are included in the model. Point estimates are presented as odds ratios (OR) with 95% confidence intervals. *PEA* Pulseless electrical activity; *STEMI* ST-Elevation myocardial infarction; *ECG* Electrocardiogram; *ROSC* Return of spontaneous circulation; CPR Cardiopulmonary resuscitation; *SD* Standard deviation; *COPD* Chronic pulmonary obstructive disease; *BMI* Body mass index; *TTM* Targeted temperature management.

To investigate the relative contribution on outcome for of all the variables in the model, we defined a subgroup of patients with linear continuous variables by empiric testing of different cutoffs for these. We found that in the group aged 42–92 years with time to ROSC 9–87 min constituting 3030 patients (76% of the total cohort), explanatory continuous variables were linear (Supplementary Fig. S2). For the patients in the subgroup analysis the adjusted OR for good outcome with circulatory shock at hospital admission and none of the defined preexisting comorbidities, was 0.65 [0.47–0.90]. The OR for the interaction between circulatory shock and preexisting hypertensive disease, as the only comorbidity, was 0.64 [0.42–0.98], indicating 36% worse odds for good outcome in patients with circulatory shock and a history of hypertension compared to circulatory shock without previous hypertension. Contrary, in circulatory shock and preexisting arrhythmia the OR for the interaction was 1.96 [1.03–3.71], indicating roughly double the odds for good outcome compared to circulatory shock alone (Fig. 3).

In sensitivity analysis of the impact of missing data, the point estimate for circulatory shock at hospital admission as a predictor for neurologic outcome at hospital discharge remained very similar to that of the imputed dataset (data not shown).

## Discussion

In this large retrospective cohort study of unconscious adult survivors of OHCA admitted to the intensive care unit, the main finding is that circulatory shock at hospital admission is independently associated with poor neurologic outcome at hospital discharge.

The use of hypotension as a surrogate definition of circulatory shock, complicates the interpretation of the underlying cause(s), since blood pressure is the product of cardiac output and systemic vascular resistance. A number of observational studies have previously investigated blood pressure levels during the early phase of critical care, leading up to the European resuscitation council 2021 guidelines recommendation of a MAP > 65 mmHg for unconscious survivors of cardiac arrest^8,10,12–14,18,19,24–34^. Seven of these studies analyzed blood pressure levels within the first hour after hospital admission^17–19,28,30,34,35^, three of them reporting conflicting evidence regarding blood pressure as an independent predictor of outcome^17,19,34^. Contrary to our findings, a single center study consisting of a mixed cohort of IHCA/OHCA cases not treated with targeted temperature management (TTM), found that MAP five minutes after ROSC was not associated with neurologic outcome in adjusted analysis^34^. Less than 15% of patients in this study had recorded 5-min MAP levels < 65 mmHg, which might reflect a lingering adrenaline effect, which could have confounded the results. In an Australian registry study, systolic arterial blood pressure < 90 mmHg at admission after OHCA was found to be independently associated with lower hospital survival only in the subgroup of patients with an initial shockable rhythm^17^. Patients in that study were aggressively resuscitated with fluid loading and prehospital vasopressors, suggesting that the relative contribution of circulatory shock on outcome might decrease in patients with the worst physiologic characteristics. In a study of a mixed cohort of IHCA/OHCA patients, a systolic arterial blood pressure < 90 mmHg within one hour of ICU admission was found to be independently associated with higher in-hospital mortality^19^. This is in line with our findings, but the authors found a higher relative contribution on outcome, possibly due patient selection and model not adjusted for time to ROSC.

Our results suggest that hypotension and/or circulatory shock may contribute to subsequent neurologic outcome. However, the association between circulatory shock (using both the pragmatic and physiological definition of circulatory shock) and neurologic outcome has, to our knowledge, not been studied. A few small observational studies report the influence of low cardiac output in the setting of the subacute phase of OHCA. Huang et al. report increased hospital survival, but no difference in neurologic outcome among patients with a cardiac index (CI) > 2.5 l/min/m^2^ at 12 h post ROSC^10^. In two other studies, no such association was found^8,9^; another study found an association between higher CI time integral and poor neurologic outcome at 28 days^11^. Two randomized controlled trials have published neutral results on surrogate markers for neurologic outcome using circulatory interventions to optimize cerebral oxygen delivery in the acute phase post OHCA^15,16^. The results of our subgroup analysis suggest an interaction between circulatory shock on admission and pre-arrest hypertensive disease. This finding is in line with previous studies^14,36^; the effect was not significant in the full cohort. The results of the exploratory subgroup analysis should be cautiously interpreted in the context of multi-testing and should be seen as hypothesis generating.

This study adds to the previous conflicting evidence that early circulatory shock in the context of OHCA is independently associated with poor neurologic outcome. Autoregulation is the innate mechanism regulating cerebral blood flow over a wide range of perfusion pressures, mitigating ischemia and hyper perfusion^37^. The lower limit of autoregulation, however, is right shifted after cardiac arrest^38^. This leaves cerebral blood flow reliant on blood pressure, rendering cerebral oxygenation vulnerable in hypotension. This process is further aggravated by cerebral hypoperfusion caused by microcirculatory injury^39^, potentially explaining our findings. Results also suggest that individually optimized blood pressure targets should be investigated in future studies, as some patients may benefit from higher post-arrest blood pressure targets. This could explain why hemodynamic interventional trials have failed to improve outcome in the context of cardiac arrest. Comparing the point estimates in our study, the relative influence of the presence of circulatory shock at the time of hospital admission on neurologic outcome at hospital discharge is minor as compared to some of the other explanatory variables in our model, specifically first documented rhythm, age, and time to ROSC. Explained variance is low, indicating the need for further studies targeting the pathophysiologic mechanism of circulatory shock in this context and how individual physiological characteristics may affect the trajectory of circulatory shock and outcomes.

## Limitations

Definitions of circulatory shock, timing and duration of circulatory shock, patient selection, sample size, TTM-levels and timing/choice of endpoints make comparison of studies cumbersome. We acknowledge a number of limiting factors in our study, the most obvious ones being: (1) The lack of a universally accepted definition of circulatory shock; (2) The sensitivity and specificity for circulatory shock with the predefined definition used in the INTCAR database has not been published. (3) Additional signs normally associated with the clinical diagnosis of circulatory shock, e.g. lactate, was not included in the INTCAR database. (4) Neurologic outcome in this study was evaluated at hospital discharge, which may be too early for complete neurologic recovery. (5) Additional outcome measures of neurologic recovery or neurologic injury, eg neuropsychiatric/cognitive testing and biomarkers indicating cerebral injury (eg. NSE, NFL and S-100), was not available in the predefined dataset. Due to the correlation between dichotomized neurologic outcome with mortality, our results also should be interpreted within the context of survival. Further, the study population represents a convenience sample and registered data were not monitored, which introduces potential bias. The influence of circulatory shock on outcome in the setting of cardiac arrest is inherently difficult because of competing risk of poor outcome due to the initial hypoxic/ischemic insult. We lack data on cause of arrest, biomarkers and physiological parameters, which could account for the low explained variance in our models. Analyses were made on a large predefined dataset, and although the sensitivity analysis over a wide range was similar to the results of the imputed dataset, there is no guarantee that missing data did not affect the results. By virtue of design, causal inferences cannot be made.

## Conclusion

Circulatory shock at hospital admission after OHCA is independently associated with poor neurologic outcome at hospital discharge.

## Supplementary Information

Supplementary Information.

## Data Availability

The data that support the findings of this study are available from corresponding author, but restrictions apply to the availability of these data, which were used under license for the current study, and so are not publicly available. Data are however available from the corresponding author upon reasonable request.
