# Supplementary material for: Influence of circulatory shock at hospital admission on outcome after out-of-hospital cardiac arrest
Source: Sci Rep. 2022 May 18;12:8293. doi: 10.1038/s41598-022-12310-5 (PMC9117194; doi:10.1038/s41598-022-12310-5)
Supplement: Supplementary file 1 — Supplementary Information. [file 41598_2022_12310_MOESM1_ESM.pdf]

Influence of circulatory shock at hospital admission on outcome after out-of-hospital cardiac arrest

*Joachim Düring[1], Martin Annborn[2], Josef Dankiewicz[3], Allison Dupont[4], Sune Forsberg[5], Hans Friberg[1], Karl B. Kern[6], Teresa L. May[7], John McPherson[8], Nainesh Patel[9], David B. Seder[7], Pascal Stammet[10], Kjetil Sunde[11], Eldar Søreide[12], Susann Ullén[13], Niklas Nielsen[2]

[1] Department of Clinical Sciences, Anesthesia & Intensive care, Lund University, Skåne University Hospital, Malmö, Sweden.

[2] Department of Clinical Sciences Lund, Anesthesia & Intensive care, Lund University, Helsingborg Hospital, Helsingborg, Sweden.

[3] Department of Clinical Sciences, Cardiology, Lund University, Skåne University Hospital, Lund, Sweden.

[4] Department of Cardiology, Northside Cardiovascular Institute, Georgia, United States.

[5] Department of Intensive Care, Norrtälje Hospital, Center for Resuscitation Science, Karolinska Institute, Sweden.

[6] Division of Cardiology Department of Medicine University of Arizona Tucson AZ.

[7] Department of Critical Care Services, Maine Medical Center, Portland, ME, United States.

[8] Vanderbilt University Medical Center, Nashville, United States.

[9] Department of Cardiology, Lehigh Valley Health Network, PA, United States.

[10] Department of intensive care medicine, Centre Hospitalier de Luxembourg, Luxembourg (LU); Faculty of Science, Technology and Medicine, University of Luxembourg, Esch-sur-Alzette, Luxembourg (LU)

[11] Department of Anesthesiology, Division of Emergencies and Critical Care, Oslo University Hospital, Oslo, Norway; Institute of Clinical Medicine, University of Oslo, Oslo, Norway.

[12] Critical Care and Anesthesiology Research Group, Stavanger University Hospital, Norway; Department of Clinical Medicine, University of Bergen, Bergen, Norway.

[13] Clinical Studies Sweden- Forum South, Skåne University Hospital, Lund, Sweden.

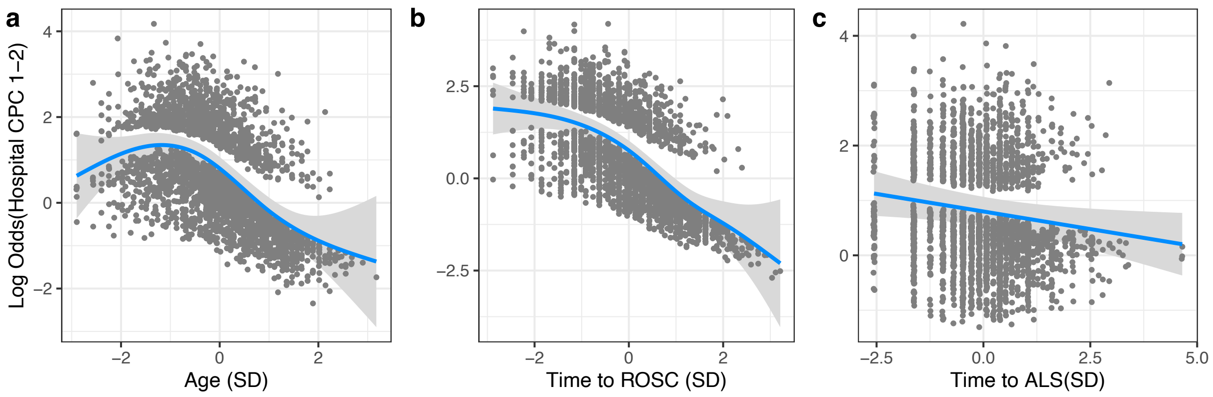

Supplementary figure S1: Association with outcome for continuous variables. Graphical illustration using multivariate generalized additive methods with smooth functions fitting continuous data to cubic restricted splines using 5 knots. Analysis was performed on the full cohort, n = 4004. Blue line illustrates the association of the log odds for good neurologic outcome at hospital discharge, defined as Cerebral Performance Category 1-2, with continuous variables used in the model. Light gray area indicating the 95% confidence band. Age (years) and time to ROSC (minutes) have been transformed to normality by ordered quantiles, time to ALS has been square root transformed. After transformation the variables have been scaled to standard deviations and centered. A nonlinear association with outcome was observed for age (a) time to ROSC (b), while time to ALS was linear (c). ROSC; Return of Spontaneous Circulation, ALS; Advanced Life Support.

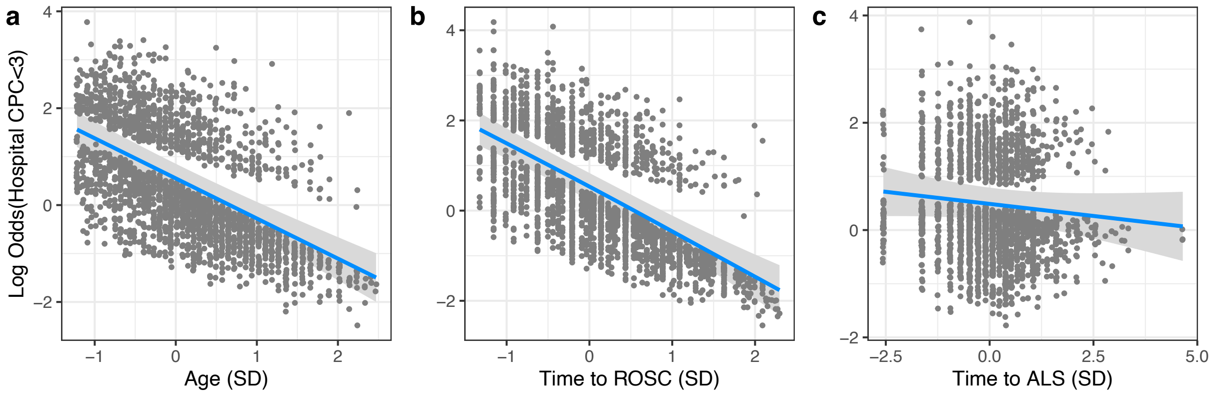

Supplementary figure S2: Association with outcome for continuous variables in subgroup analysis. Graphical illustration using multivariate generalized additive methods with smooth functions fitting continuous data to cubic restricted splines using 5 knots. Analysis was performed in a subgroup of patents, n= 3003 (76% of full cohort) aged 42 - 92 years, with time to ROSC 9 - 87 minutes. Blue line illustrates the association of the log odds for good neurologic outcome at hospital discharge, defined as Cerebral Performance Category 1-2,

with continuous variables used in the model. Light gray area indicating the 95% confidence band. To estimate the relative contribution on outcome, continuous variables are shown as standard deviations of the original range: Age (years) and time to ROSC (minutes) have been transformed to normality by ordered quantiles, time to ALS has been square root transformed. After transformation the variables have been scaled to standard deviations and centered. A linear association with outcome is shown for age (a) time to ROSC (b) age and time to ALS (c) in this subgroup. ROSC; Return of Spontaneous Circulation, ALS; Advanced Life Support.
